# PTPRO-related CD8^+^ T-cell signatures predict prognosis and immunotherapy response in patients with breast cancer

**DOI:** 10.3389/fimmu.2022.947841

**Published:** 2022-08-08

**Authors:** Hongmei Dong, Chaoyu Xie, Zhimeng Yao, Ruijun Zhao, Yusheng Lin, Yichen Luo, Shuanglong Chen, Yanfang Qin, Yexi Chen, Hao Zhang

**Affiliations:** ^1^ Institute of Precision Cancer Medicine and Pathology, School of Medicine, Jinan University, Guangzhou, China; ^2^ Department of General Surgery, The First Affiliated Hospital of Jinan University, Jinan University, Guangzhou, China; ^3^ Department of Breast Surgery, The Third Hospital of Nanchang, Nanchang, China; ^4^ Department of Hematology, University of Groningen, University Medical Center Groningen, Groningen, Netherlands; ^5^ Graduate School, Shantou University Medical College, Shantou, China; ^6^ Department of Pathology, School of Medicine, Jinan University, Guangzhou, China; ^7^ Department of General Surgery, The Second Affiliated Hospital of Shantou University Medical College, Shantou, China; ^8^ Institute of Precision Cancer Medicine and Pathology, School of Medicine, and Minister of Education Key Laboratory of Tumor Molecular Biology, Jinan University, Guangzhou, China

**Keywords:** breast cancer, PTPRO, prognosis, immune cell, TILs, immunotherapy response indicator, PTPRO-related CD8^+^ T-cell marker genes signature

## Abstract

**Background:**

Poor immunogenicity and extensive immunosuppressive T-cell infiltration in the tumor immune microenvironment (TIME) have been identified as potential barriers to immunotherapy success in “immune-cold” breast cancers. Thus, it is crucial to identify biomarkers that can predict immunotherapy efficacy. Protein tyrosine phosphatase receptor type O (PTPRO) regulates multiple kinases and pathways and has been implied to play a regulatory role in immune cell infiltration in various cancers.

**Methods:**

ESTIMATE and single-sample gene set enrichment analysis (ssGSEA) were performed to uncover the TIME landscape. The correlation analysis of PTPRO and immune infiltration was performed to characterize the immune features of PTPRO. Univariate and multivariate Cox analyses were applied to determine the prognostic value of various variables and construct the PTPRO-related CD8^+^ T-cell signatures (PTSs). The Kaplan–Meier curve and the receiver operating characteristic (ROC) curve were used to estimate the performance of PTS in assessing prognosis and immunotherapy response in multiple validation datasets.

**Results:**

High PTPRO expression was related to high infiltration levels of CD8^+^ T cells, as well as macrophages, activated dendritic cells (aDCs), tumor-infiltrating lymphocytes (TILs), and Th1 cells. Given the critical role of CD8^+^ T cells in the TIME, we focused on the impact of PTPRO expression on CD8^+^ T-cell infiltration. The prognostic PTS was then constructed using the TCGA training dataset. Further analysis showed that the PTS exhibited favorable prognostic performance in multiple validation datasets. Of note, the PTS could accurately predict the response to immune checkpoint inhibitors (ICIs).

**Conclusion:**

PTPRO significantly impacts CD8^+^ T-cell infiltration in breast cancer, suggesting a potential role of immunomodulation. PTPRO-based PTS provides a new immune cell paradigm for prognosis, which is valuable for immunotherapy decisions in cancer patients.

## Introduction

Immunotherapy emerged as a new promising therapeutic approach for breast cancer, especially in triple-negative breast cancer (TNBC), and has been approved in combination with chemotherapy, radiation, and targeted therapeutics (1, 2). However, most types of cancers are recognized as “cold” tumors characterized by poor immunogenicity and T-cell dysfunction in the tumor immune microenvironment (TIME), which have been considered obstacles to immunotherapy efficacy. TNBC is more responsive to immunotherapy than other breast cancer subtypes as it has more tumor-infiltrating lymphocytes (TILs), higher expression of programmed cell death ligand-1 (PD-L1) on tumor and immune cells, and a higher number of non-synonymous mutations (3, 4). Although TNBC has a greater response rate to immune checkpoint inhibitors (ICIs) than other breast cancer subtypes, monotherapy response rates remain extremely low, with only 5% of unselected patients responding and 23% of PD-L1-positive patients responding (5, 6). Currently, three validated biomarkers (mismatch repair deficiency, PD-L1, and TILs) have been adopted for selecting patients and predicting clinical benefit from single-agent ICIs (2, 7). However, the coordination between cancer cells and the immune system in breast cancer is a dynamic, evolving, and complex biological process, which needs to discover more comprehensive immune-related biomarkers (2). Therefore, identifying effective indicators of immunotherapy response is critical for immunotherapy in breast cancers.

Tumor-infiltrating CD8^+^ T cells are associated with the clinical benefit of ICI therapy in many cancers (8). However, given that CD8^+^ T cells become dysfunction states (tolerance, ignorance, anergy, and exhaustion, respectively) during cancer development, most patients are unable to maintain a long-term response to immunotherapy (9). Currently, there is not any effective indicator to predict which patients will respond, even though much effort has been made. The mechanisms that determine clinical response to immunotherapy remain largely unknown. Emerging technologies (such as spatially resolved transcriptomics, bulk and single-cell transcriptomics, single-nucleus RNA-seq, and epigenetic profiling) have allowed us to initially characterize the features of CD8^+^ T-cell heterogeneity and the regulatory mechanisms of CD8^+^ T-cell differentiation and dysfunction (9, 10). More recently, CD8^+^ tissue-resident memory T (T_RM_) cells were revealed by single-cell RNA sequencing (scRNA-seq) on breast cancer T cells (11). These T-cell subsets are characterized by high expression levels of immune checkpoint molecules and effector proteins and contribute to patient prognosis and response to anti-PD-1 therapy in TNBC (11, 12). The scRNA-seq has provided important insights into the features of T_RM_ cells, and hence can aid in the development of effective immunotherapy targeting T cells; however, the molecular basis of T-cell dysfunction states in breast cancer remains elusive (11). It is necessary to refine the indicators that allow for the identification of CD8^+^ T-cell phenotypes and to explore the regulatory mechanisms of CD8^+^ T cells, especially in other breast cancer subtypes except for TNBC (11, 13, 14).

The protein tyrosine phosphatases (PTPs) catalyze the dephosphorylation of specific target protein tyrosine kinases (PTKs) as a common means of regulating cellular signal transduction and play an important regulatory role in immune cell signaling (15, 16). Protein tyrosine phosphatase receptor type O (PTPRO), a member of the PTP family, has been reported that it can function as a tumor suppressor and prognostic factor in various cancers (16–19). Furthermore, downregulation of PTPRO by aberrant hypermethylation in various cancer types, including lung cancer, hepatocellular carcinoma (HCC), breast cancer, esophageal cancer, and leukemia, suggests that it may be a therapeutic candidate for epigenetic therapy (20–24). Additionally, given the regulatory functions of PTPRO in immune cells, we and other groups have begun to focus on the roles of PTPRO in tumor immunity (16). Our recent study indicated that tumor-derived exosomal PTPRO could ameliorate the immunosuppressive tumor microenvironment (ITM) and inhibit breast tumor cell metastasis by resetting tumor-associated macrophages (TAMs) (25). We also found that PTPRO could predict patient prognosis and be significantly associated with the immune infiltrate of clear cell renal cell carcinoma (ccRCC) (26). Another study further confirmed that PTPRO is a therapeutic target and promotes the infiltration of immune cells including CD8^+^ T cells, macrophages, dendritic cells, and neutrophils in pancreatic cancer (27). However, little is known about PTPRO’s role in the immunotherapy response in breast cancer. In this study, we provide evidence that PTPRO as a potential immune indicator and PTPRO-related CD8^+^ T-cell signatures (PTSs) can be used to predict prognosis and immunotherapy response in breast cancer patients.

## Materials and methods

### Data collection and reprocessing

The RNA-seq data contained 130 patient samples (from the series GSE65194), and 251 patient samples (from the series GSE3494) were obtained from the Gene Expression Omnibus (GEO) database. ScRNA-seq profiling of two primary TNBCs was obtained from GSE110686. The Cancer Genome Atlas (TCGA) breast cancer RNA-seq profiling [in the form of fragments per kilobase million (FPKM)], mutation data, and corresponding clinicopathological data were obtained from TCGA database. RNA-seq expression data of 3,273 breast cancer samples (GSE96058) in the form of log_2_ (FPKM + 0.1) and corresponding clinicopathological characteristics were obtained from the GEO database. RNA-seq profiling of 1,904 breast cancer samples (METABRIC) and corresponding clinicopathological characteristics were derived from the cBioPortal. The profiling in the form of FPKM or log_2_ (FPKM + 0.1) was converted into transcripts per kilobase million (TPM) values and was further log_2_-transformed [log_2_ (TPM + 0.1)] (28).

### Associations between PTPRO and the infiltration of immune cells

The “ESTIMATE” R package was utilized to calculate the immune scores, stromal scores, and ESTIMATE scores, respectively, which can be used to evaluate the abundance of immune cells and stromal cells in the breast cancer microenvironment. The infiltration and function of immune cells were quantified by single-sample gene set enrichment analysis (ssGSEA) *via* the “gsva” R package. Among the GSE65194 and GSE3494 datasets with complete gene expression data, samples based on PTPRO expression were divided into high (upper 50%) and low (lower 50%) expression groups, respectively.

### Patients

Breast cancer patients (*n* = 30) were obtained from the Cancer Hospital affiliated to Shantou University Medical College, undergoing surgical treatment at the Department of Surgery, during the period from 2010 to 2013. All patients received primary treatment by surgery followed by adjuvant radiotherapy, chemotherapy, or hormone therapy. The mean age of the patients was 50 years (range: 20–75 years). The clinical research protocols of this study were reviewed and approved by the Ethics Committee of Shantou University Medical College (IRB serial number: #04-070). Written informed consent was obtained from the patients in accordance with the principles expressed in the Declaration of Helsinki.

### Immunohistochemical analysis

Immunohistochemistry (IHC) staining was performed as previously described (18, 19). In brief, 4-µm sections from representative breast cancer tumor tissue were cut from formalin-fixed paraffin-embedded specimens and underwent deparaffinization, rehydration, endogenous peroxidase blocking, and antigen retrieval. The following primary antibodies were used: PTPRO (Cat# sc-365654, Santa Cruz, CA, USA), and CD8 (Cat# ab101500, Abcam, Cambridge, UK). Furthermore, the primary antibodies were incubated at 4°C overnight. Then, the sections were incubated with horseradish peroxidase (HRP)-conjugated secondary antibodies at room temperature for 1 h, followed by color development with 3,3′-diaminobenzidine (DAB) substrate kit (DAKO, Glostrup, Denmark). The nuclei were counterstained with hematoxylin.

The percentage of PTPRO expression in the tumor cells was scored using the following scales: 0, negative; 1, ≤10%; 2, 11%–50%; 3, 51%–75%; and 4, >75%. The intensity of staining was scored using the following scales: 1, weak staining; 2, moderate staining; and 3, strong staining. The percentage (*P*) and intensity (*I*) of the cytoplasm or membrane expression were multiplied to generate a numerical score (*S* = P*I), which was modified from previous studies (19).

### Identification of PTPRO-related CD8^+^ T-cell marker genes

The “Seurat” and “SingleR” packages were used to analyze the scRNA-seq data (29). Cells with a number of detectable genes less than 200 and genes detected less than 3 cells were moved. We performed principal component analysis (PCA) with 1,500 variable genes to cluster the single cells followed by t-distributed stochastic neighbor embedding (t-SNE) with the first 20 PCA components using the RunPCA and RunTSNE functions, respectively. The “SingleR” R package was utilized for cell-type annotation, which works by comparing the transcriptome of every single cell with reference datasets. Absolute log_2_ fold change > 0.5 and an adjusted *P* < 0.05 were used to define the marker genes. After that, expression correlation assays between PTPRO with CD8^+^ T-cell marker genes were conducted using Spearman’s coefficient correlation among the TCGA dataset.

### Construction and validation of a prognostic signature in breast cancer

The cases from the TCGA breast cancer datasets were included for the construction of the prognostic model. Univariate Cox analysis of overall survival (OS) was complemented to screen PTPRO-related CD8^+^ T-cell marker genes with prognostic values. The multivariate Cox proportional hazards model was established using statistically significant genes from the univariate Cox analysis. The independent prognostic factors were evaluated by the multivariate Cox proportional hazard regression model. The risk scores of the patients were established as follows: risk score = *β*_1_ *x*_1_ + *β*_2_ *x*_2_ +… + *β_i_x_i_*. In this formula, *x_i_* was the expression value of each gene obtained from the prognostic model, while *β_i_* was the corresponding coefficient. The Kaplan–Meier method was used for survival analysis, and the samples were divided into high and low groups according to the median value of the risk score. The prognostic model’s prediction capability was quantified by the receiver operating characteristic (ROC) curve using the R-package “timeROC” (30).

### Construction of the nomogram

Based on the results of the multivariable Cox regression analysis, a nomogram integrating clinicopathological parameters (including age, stage, TNM stage, and risk score) was developed through the R package “rms.” All of these points are added up for each individual to generate a total point, which can predict the 1-, 3-, and 5-year survival probability of breast cancer patients. The calibration curve was plotted to evaluate the nomogram’s discrimination. The predictive accuracy of the nomogram was quantified by the concordance index (C-index).

### Genomic and clinical datasets with anti‐PD‐L1 therapy

A urothelial carcinoma cohort (298 cases with complete clinical characteristics) that received the anti‐PD‐L1 therapy from the IMvigor210 cohort was used to analyze and explore the predictive accuracy of the prognostic signature (31). RNA-seq profiling and the corresponding clinicopathological characteristics were obtained from the Creative Commons 3.0 License. The count value was transformed into the log_2_ (TPM + 1) value.

### Statistical analysis

Student’s *t*-tests were used to compare the normal distributions between two groups, and the Wilcoxon rank-sum test was performed to compare the non-normal distributions between two groups. The prognostic factors were evaluated *via* the univariate and multivariate Cox regression models, and further construction of the prognostic model was established through the “survival” and “survminer” R packages. The multivariable analysis model was constructed with variables with a *P*-value < 0.15 in the univariable analysis (32). Then, in the multivariate model, 11 candidate genes (*P*-value < 0.15) were selected because correlations can play an important role to build better prognostic models (33). In the TCGA, METABRIC, GSE96058, and IMvigor210 datasets, patients were grouped according to high or low risk based on median risk scores (34). Survival analysis was measured using the Kaplan–Meier method. Then, the log-rank test was performed to analyze the significance of disparity. The “timeROC” R package was performed to evaluate the accuracy of the prognostic model. The “ClusterProfiler,” “org.Hs.eg.db,” and “enrichplot” R packages were utilized for the GSEA analysis. The statistical analysis was performed using R software (version 4.1.0). *P-*value <0.05 was considered to be statistically significant.

## Results

### The role of PTPRO on breast cancer tumor microenvironment

**Figure 1:**
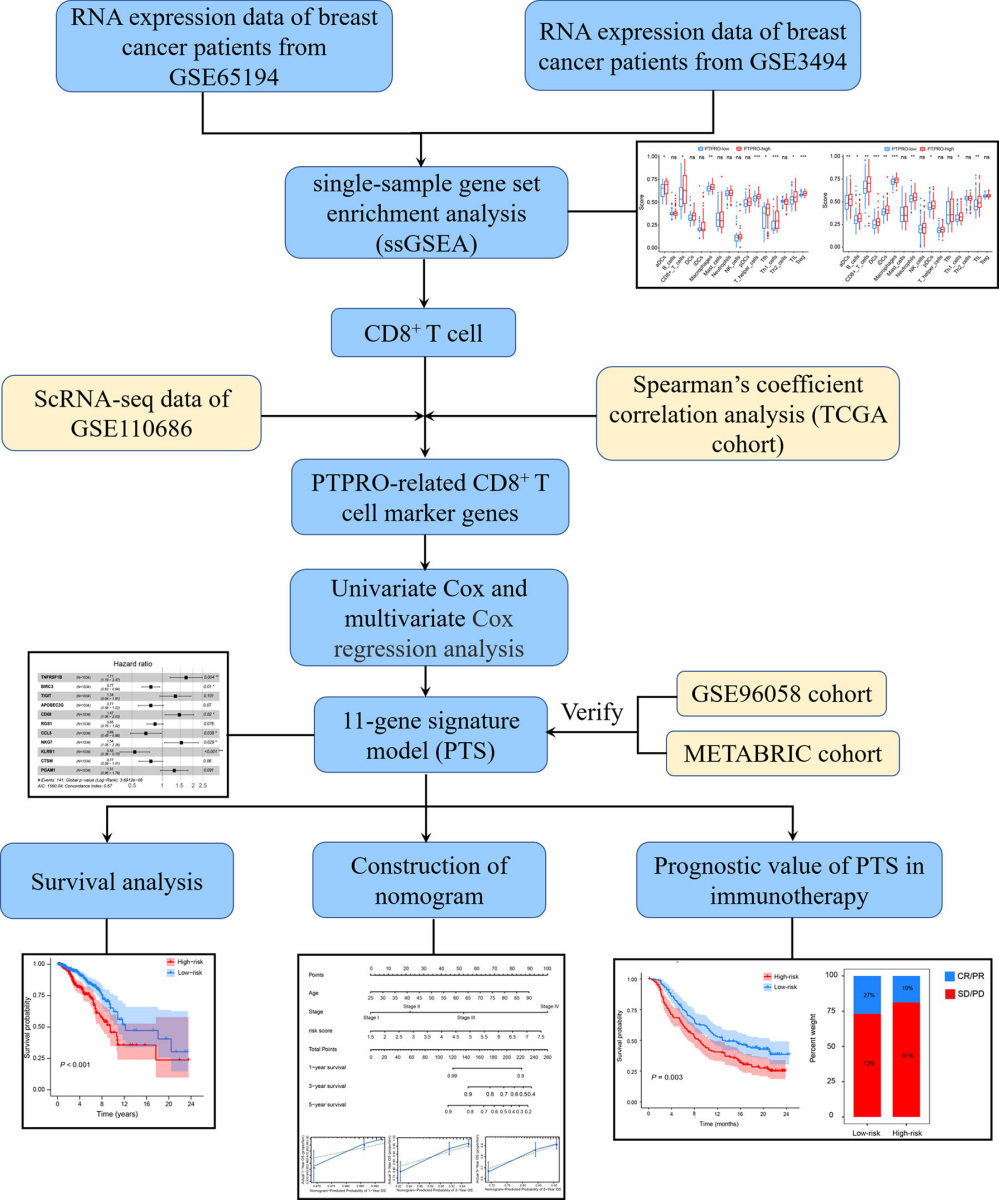
The flowchart of signature construction and verification.

**Figure 2:**
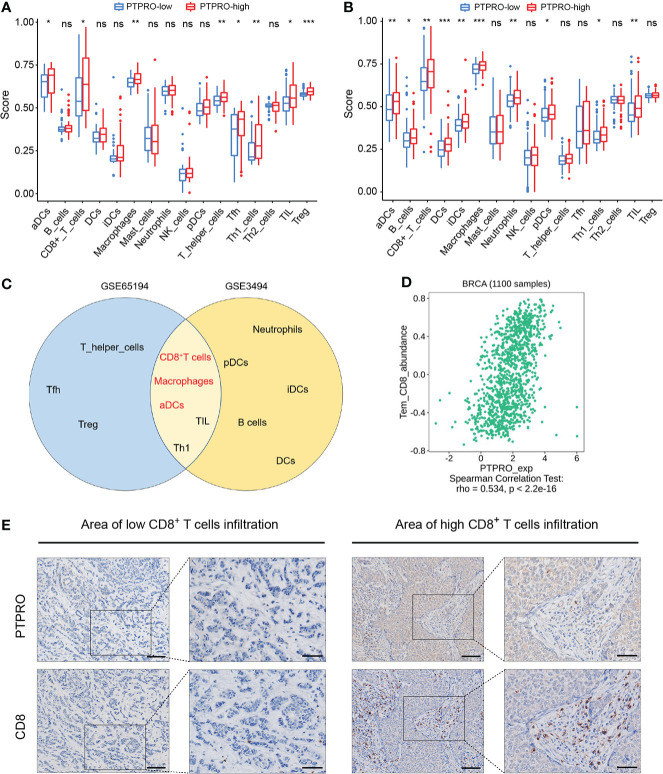
Characterization of protein tyrosine phosphatase receptor type O (PTPRO) in breast cancer tumor microenvironment. Comparison of the ssGSEA scores between the PTPRO-high group and the PTPRO-low group in the GSE65194 **(A)** and GSE3494 **(B)** cohorts. **(C)** Overlapped immune cell types correlated with PTPRO expression in the two cohorts. **(D)** The dot plots displayed the correlations between PTPRO expression and the infiltration pattern of CD8^+^ T cells in TISIDB. **(E)** Representative IHC staining indicates higher PTPRO levels correlated with increased CD8^+^ T-cell infiltrates in human breast cancer. Scale bars: 100 μm (left panel), 50 μm (right panel). ns, not significant; *P < 0.05; **P < 0.01; ***P < 0.001 by Student’s *t*-test.

As shown in **Figure 1**, the TCGA cohort was used as the training cohort, with 1,034 patients having a survival time of more than 30 days included. The METABRIC and GSE96058 cohorts were viewed as the external validation cohorts, consisting of 1,904 and 3,069 patients with survival data, respectively. To investigate the influence of PTPRO on the TIME, we explored the correlation between PTPRO expression and immune cell distribution. Through ESTIMATE, we found that samples with low PTPRO had significantly lower immune scores than the high PTPRO samples (**Supplementary Figure S1A**, GSE65194; **Supplementary Figure S1B**, GSE3494). We further evaluated the correlation between PTPRO and immune status and found that the enrichment scores for 16 immune cell types and 13 immune-related pathways were lower in the PTPRO-low group than in the PTPRO-high group, indicating that patients in the PTPRO-high group may have better immune status and immune function (**Figure 2A**, **Supplementary Figure S1C**, GSE65194; **Figure 2B**, **Supplementary Figure S1D**, GSE3494). Notably, in two GEO cohorts, five immune cell types, namely, CD8^+^ T cells, macrophages, activated dendritic cells (aDCs), TILs, and Th1, were found to be significantly more abundant in the PTPRO-high group (**Figure 2C**). It is well known that CD8^+^ T cells are the central subpopulation of cytotoxic T cells, which are primarily responsible for eliminating tumor cells (35). Given the importance of CD8^+^ T-cell infiltration in the TIME, the relationship between CD8^+^ T-cell infiltration levels and PTPRO expression was further investigated. The results showed that PTPRO expression was significantly positively related to CD8^+^ T-cell infiltration levels in the TISIDB database (**Figure 2D**). Furthermore, we performed the IHC staining assay to analyze PTPRO and CD8 expression in 30 human breast cancer tissues (**Figure 2E**). Tumor infiltration of CD8^+^ T cells was significantly higher in tumors with higher PTPRO expression than in tumors with low PTPRO expression (*r* = 0.914; *P* < 0.001) (**Supplementary Figure S1E**). Collectively, these results suggest that PTPRO plays an essential role in mediating the reprogramming of TIME, thereby suppressing tumor progression.

### Construction of prognostic PTPRO-related PTS

**Figure 3:**
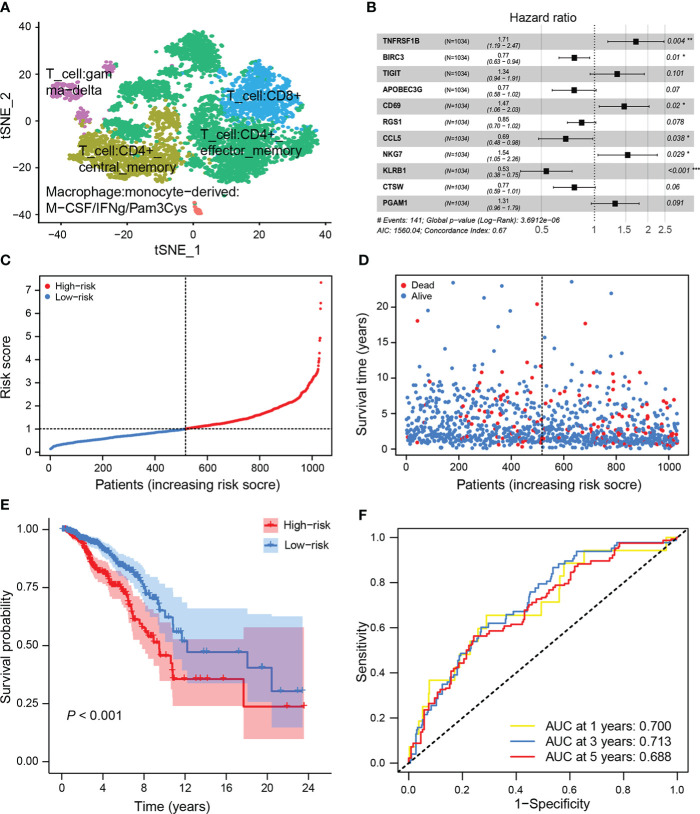
Construction of the PTPRO-related CD8^+^ T-cell signature (PTS) in the training set. **(A)** t-SNE plot depicted various cell types. **(B)** The prognostic signature was developed by multivariate analysis of candidate genes that were associated with the overall survival (OS) of breast cancer patients in the training set. **(C)** Breast cancer patients in the training set were divided into high-risk and low-risk groups based on the median value of the risk score. **(D)** Breast cancer patients’ survival status and risk score distribution in the training set. **(E)** Kaplan–Meier curve analysis of OS between the high-risk and low-risk groups in the training set. **(F)** ROC curves of the risk score to predict the 1-, 3-, and 5-year OS in the training set.

Given that TIME’s immune profile, including CD8^+^ T-cell-related genes, has been shown to correlate with prognosis (36, 37), and based on PTPRO’s regulatory role in promoting CD8^+^ T-cell infiltration, we further investigated the association between CD8^+^ T-cell-related genes and PTPRO. By analyzing the scRNA-seq data from the GSE110686 cohort, 127 CD8^+^ T-cell marker genes were confirmed (**Figure 3A**, **Supplementary Figure S2A**). Among them, 56 CD8^+^ T-cell-related genes were subsequently identified to be significantly associated with PTPRO (filtering thresholds were set as *R* > 0.3, *P* < 0.05) in the TCGA dataset (**Supplementary Table 1**). Next, the TCGA breast cancer dataset was used as the training cohort to evaluate the prognostic value of the above 56 genes. A total of 31 genes (*SRGN*, *SERPINB9*, *ICOS*, *CD74*, *TNFRSF1B*, *CXCR6*, *BIRC3*, *TIGIT*, *CTLA4*, *APOBEC3G*, *TRAC*, *CD69*, *SIRPG*, *GZMA*, *CD52*, *CST7*, *RGS1*, *CD8A*, *GZMK*, *SPOCK2*, *ZNF683*, *GBP2*, *CCL5*, *HCST*, *NKG7*, *KLRB1*, *CTSW*, *CD8B*, *TRGC2*, *PGAM1*, and *PIM2*) were found to contribute to the OS as revealed by the univariate Cox proportion hazards regression analysis (**Supplementary Figure S2B**). A multivariate Cox regression analysis revealed that 11 candidate genes were determined and subsequently used to create a prognostic signature (i.e., PTS) (**Figure 3B**). The PTS risk score for predicting prognosis was calculated using the formula: PTS risk score = *TNFRSF1B* expression × (0.5385) + *BIRC3* expression × (−0.2625) + *TIGIT* expression × (0.2949) + *APOBEC3G* expression × (−0.2651) + *CD69* expression × (0.3853) + *RGS1* expression × (−0.1672) + *CCL5* expression × (−0.3752) + *NKG7* expression × (0.4308) + *KLRB1* expression × (−0.6283) + *CTSW* expression × (−0.2624) + *PGAM1* expression × (0.2686) (**Supplementary Table 2**).

The corresponding PTS risk scores were calculated for each breast cancer patient in the training cohort (**Figure 3C**). The median value of the PTS risk score was used as the cutoff value to divide patients into low-risk (*n* = 517) and high-risk (*n* = 517) groups. The distribution of PTS risk score and patient survival status revealed that patients with high risk died sooner than those with low risk (**Figure 3D**). Consistently, patients with high risk had a significantly shorter OS than patients with low risk (*P* < 0.001, **Figure 3E**). ROC analysis was performed to interpret the predictive performance of PTS risk score, and the results showed that the AUCs for 1-, 3-, and 5-year OS were 0.700, 0.713, and 0.688, respectively (**Figure 3F**). Furthermore, GSEA showed that immune-related gene sets were enriched in patients with low-risk score (**Supplementary Figure S2C**). Therefore, our findings suggest that PTS risk score has a high specificity and sensitivity for predicting the OS of breast cancer patients.

### Validation of the prognostic value of PTS risk score

**Figure 4:**
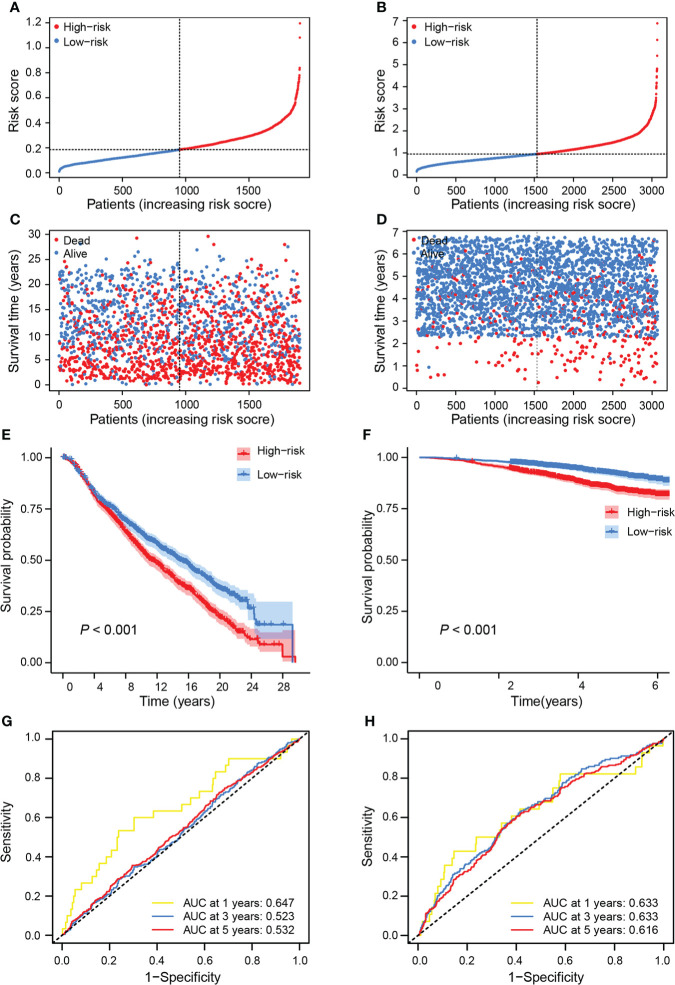
Validation of the prognostic value of risk score in independent cohorts. Breast cancer patients in the training set were separated into high-risk and low-risk groups based on the median value of risk score in the METABRIC cohort **(A)** and the GSE96058 cohort **(B)**. Breast cancer patients’ survival status and risk score distribution in the METABRIC cohort **(C)** and the GSE96058 cohort **(D)**. Kaplan–Meier curves of OS between the high-risk and low-risk groups in the METABRIC cohort **(E)** and the GSE96058 cohort **(F)**. ROC curves showed the performance of risk score in predicting the 1-, 3-, and 5-year OS in the METABRIC cohort **(G)** and the GSE96058 cohort **(H)**.

To evaluate the robustness of the PTS, we tested its predictive power in two independent validation cohorts from the METABRIC and GSE96058 datasets. Risk scores were calculated for patients in two cohorts using the same formula generated in the training cohort (**Figures 4A, B**). Similar to the training cohort, patients with high risk died sooner than those with low risk (**Figures 4C, D**). Patients were then separately classified into high-risk groups (METABRIC, *n* = 951; GSE96058, *n* = 1,534) and low-risk groups (METABRIC, *n* = 952; GSE96058, *n* = 1,535) based on the median values of the risk score. Patients in the low-risk group had a significantly better OS than those with high risk (METABRIC, *P* < 0.001; GSE96058, *P* < 0.001) (**Figures 4E, F**). Moreover, the AUCs for 1-, 3-, and 5-year OS of this classifier were 0.647, 0.523, and 0.532 in METABRIC and 0.633, 0.633, and 0.616 in GSE96058, respectively (**Figures 4G, H**), further confirming the prognostic role of the PTS.

### Independence of the PTS risk score from other clinical characteristics

**Figure 5:**
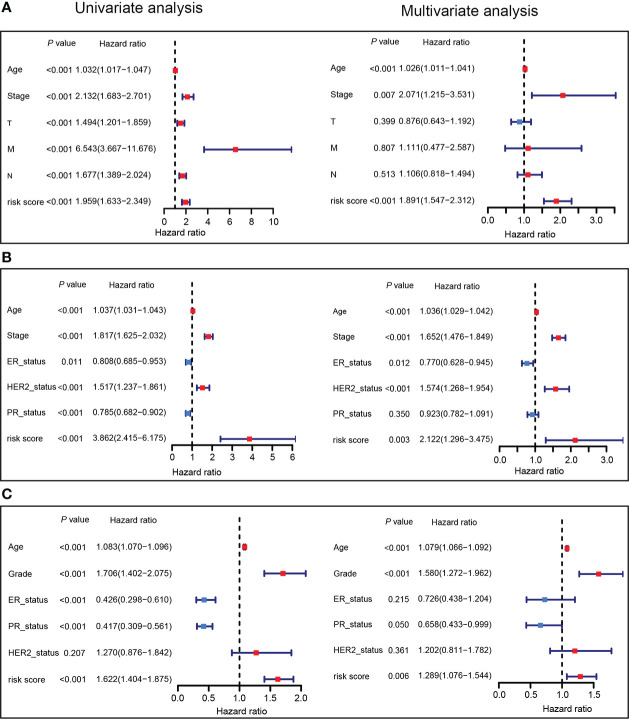
The prognostic values of PTS risk score in breast cancer. Univariate and multivariate Cox regression analyses of the PTS risk score in the TCGA training dataset **(A)**, METABRIC validation dataset **(B)**, and GSE96058 validation dataset **(C)** regarding OS.

In order to better understand the utility of the PTS in predicting the OS of breast cancer patients, the risk score and clinical features were integrated into the univariate and multivariate analyses (**Figure 5**). The multivariate analysis revealed that low-risk score remained significantly associated with favorable OS even after adjusting for other clinical characteristics. The risk score for OS was 1.891 (95% CI = 1.547–2.312, *P* < 0.001; **Figure 5A**) in the TCGA training set, 2.122 (95% CI = 1.296–3.475, *P *= 0.003; **Figure 5B**) in the METABRIC validation set, and 1.289 (95% CI = 1.076–1.544, *P *= 0.006; **Figure 5C**) in the GSE96058 validation set. Together, these data strongly demonstrate that the prognostic signature derived from PTPRO-associated immunomodulators was an independent predictor of OS in breast cancer patients.

### Construction and evaluation of a prognostic nomogram

Based on the findings of multivariate analysis, we constructed a nomogram model employing clinical factors, such as risk score, age, and stage. By calculating the score of the aforementioned variables for each patient, we can predict the individuals’ 1-, 3-, and 5-year OS probability (**Supplementary Figure S3A**). The calibration curves further revealed that the nomogram performed well in predicting breast cancer patients’ survival (**Supplementary Figures S3B–D**). The C-index of the nomogram was 0.747, which shows that it has a good capacity for discrimination.

### The prognostic value of PTS in patients with anti-PD-L1 therapy

**Figure 6:**
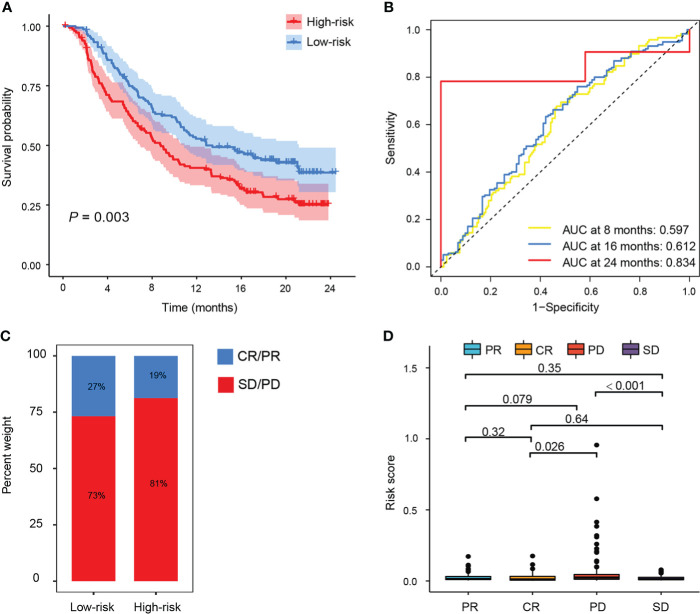
The prognostic value of PTS in patients with anti-PD-L1 therapy. **(A)** Kaplan–Meier curves of OS between the high-risk and low-risk groups in the IMvigor210 cohort. **(B)** ROC curves showed the performance of the risk score in predicting the 8-, 16-, and 24-month OS in the IMvigor210 cohort. **(C, D)** Risk score in patients with different responses to PD-1 treatment [complete response (CR), progressive disease (PD), partial response (PR), and stable disease (SD)].

To investigate the potential clinical efficacy of PTS in immunotherapy, we examined the distribution of checkpoint-related genes (*LAG3*, *HAVCR2*, *PDCD1LG1*, *IDO1*, *TIGIT*, *PDCD1*, *PD-L1*, and *CTLA-4*) and tumor mutation burden (TMB) in different PTS subgroups and found that *LAG3*, *PDCD1LG1*, *IDO1*, *TIGIT*, *PDCD1*, *PD-L1*, and *CTLA-4* were upregulated in patients with low risk in the TCGA training set (**Supplementary Figure S4A**), while TMB was higher in patients with high risk (**Supplementary Figure S4B**). Furthermore, we evaluated the predictive value of TMB by ROC analysis in the IMvigor210 cohort (urothelial carcinoma dataset), and we did observe that TMB does not outperform at a predictive advantage (**Supplementary Figure S4C**). Since anti-PD-L1 immunotherapy has emerged as a promising anticancer treatment (38), we next investigated the prognostic value of the risk score for immunotherapy in the IMvigor210 cohort of patients treated with anti-PD-L1. Patients with high risk who received atezolizumab had significantly shorter OS than patients with low risk (**Figure 6A**). Moreover, the AUCs for the 8-, 16-, and 24-month OS of this classifier were 0.597, 0.612, and 0.834 in the IMvigor210 cohort (**Figure 6B**), respectively. Patients with low risk had better immunotherapeutic responses (**Figures 6C, D**). Therefore, rather than TMB, the predictive value of PTS in immunotherapy may benefit from the upregulation of the checkpoint-related genes.

## Discussion

Here, we found that phosphatase PTPRO exhibits potential as an immune modulator, and PTPRO-based PTS is an independent prognostic indicator for prognosis and associated with immunotherapeutic responses. We first used ESTIMATE and ssGSEA to determine whether PTPRO expression is associated with the levels of CD8^+^ T-cell infiltration in breast cancer immune infiltrates. Then, using scRNA-seq data, we identified 56 CD8^+^ T-cell-related genes that were significantly associated with PTPRO. Furthermore, 11 candidate genes (*TNFRSF1B*, *BIRC3*, *TIGIT*, *APOBEC3G*, *CD69*, *RGS1*, *CCL5*, *NKG7*, *KLRB1*, *CTSW*, and *PGAM1*) were identified and used to build the risk model. Finally, the PTS-based risk score was used to predict prognosis and immunotherapeutic response, and it performed well in multiple validation datasets.

CD8^+^ T cells are one of the major effector cells in immunotherapy. However, when T cells are exposed to cancer antigens repeatedly, they differentiate into dysfunctional states (39, 40). Furthermore, T-cell receptor (TCR)-mediated signaling pathways are required for the establishment and progression of T-cell dysfunction (39, 40). Earlier studies have already proven that coordinated interactions between PTKs and PTPs play a key role in TCR-mediated signaling and then affect immune responses (41). Additionally, PD-L1-mediated immunosuppression is controlled largely by the activation of EGFR, MEK/ERK, NF-κB, PI3K/Akt, COX2/mPGES1/PGE2, JAK/STAT1, or JAK/STAT3 pathways, some of which are regulated by PTPRO (16, 42, 43). Therefore, our previous and other findings suggest that PTPRO, as an immunosuppressor, regulates immune infiltrates, shedding new light on immunotherapy (26, 27, 44). In this study, 11 PTPRO-related CD8^+^ T-cell marker genes were chosen to construct a PTPRO-based risk score. Our results showed that patients with low-risk scores had a significantly longer OS than those with high-risk scores in the METABRIC and GSE96058 datasets. Moreover, we established a prognostic nomogram based on the risk score and several important clinical variables for predicting individuals’ survival probability. The calibration curves revealed a higher consistency between the actual and predicted values for 1-, 3-, and 5-year OS.

We also investigated the prognostic value of risk score in anti-PD-L1 therapy to see if it can accurately predict the potential clinical efficacy of immunotherapy. We found that checkpoint-related genes (*LAG3*, *PDCD1LG1*, *IDO1*, *TIGIT*, *PDCD1*, *PD-L1*, and *CTLA-4*) were upregulated in patients with low risk in the TCGA training set. High TMB is associated with longer survival in patients treated with ICIs in multiple cancer types (45). We found that patients in the high-risk group had higher TMB levels in this study. Furthermore, in the IMvigor210 cohort, TMB had a poor predictive value. According to a recent study, high TMB only predicts PD-L1 blockade responsiveness in approximately 25% of several cancer types where high TMB correlates with CD8^+^ T-cell infiltration of the tumor (46). Numerous studies have shown that high TMB does not correlate with CD8^+^ T-cell infiltration and overall response rates (ORR) to ICIs in glioma, TNBC and prostate cancer (47). Due to the lack of broad ICI approval, a biomarker to optimize patient selection is most urgently needed. Furthermore, we found that the predictive value of risk score in immunotherapy response was validated in the IMvigor210 cohort, that is, a high-risk score predicted poorer survival and a poor response to immunotherapy. As a result of our findings, the predictive value of PTS in immunotherapy may benefit from increased expression of checkpoint-related genes rather than TMB. With technological advancements, a large number of high-dimensional databases and bioinformatics tools will emerge in the future, and PTPRO-based PTS warrants further extension and investigation. Furthermore, this is a retrospective study based on omics data, which requires additional experimental validation, particularly the regulatory effect of PTPRO on CD8^+^ T-cell markers or immune infiltration.

In summary, we found that PTPRO may play a role in antitumor immunity regulation. The immune indicator PTPRO-based PTS-related risk score can pre-evaluate the response to immunotherapy. We conclude that patients with low-risk breast cancer, as defined by high CD8^+^ T-cell infiltration and elevated expression of checkpoint-related genes, should have a better prognosis and clinical benefit from either monotherapy or combined immunotherapy.

## Data availability statement

The datasets presented in this study can be found in online repositories. The names of the repository/repositories and accession number(s) can be found in the article/**Supplementary Material**.

## Ethics statement

The studies involving human participants were reviewed and approved by the Ethics Committee of Shantou University Medical College (IRB serial number: # 04–070). The patients/participants provided their written informed consent to participate in this study.

## Author contributions

HZ and HD conceived and designed the study. CX, ZY, YSL, and SC performed the data analysis and wrote the manuscript. YCL, YQ, YC, and RZ collected the data and revised the manuscript. All authors contributed to the work and approved it for publication.

## Funding

The work was supported by grants from the National Natural Science Foundation of China (81802404 to HD); Natural Science Foundation of Guangdong Province of China (2021A1515012522, 9151018004000000, and 2022A1515010925 to HZ; 2021A1515011028 and 2022A1515011739 to HD); Science and Technology Planning Project of Guangdong Province of China (2019A030317024 to HZ); Special Project on the Integration of Industry, Education and Research of Guangdong Province (2011A090100024 to HZ); Jinan University Innovation and Entrepreneurship Fund for College Students (202010559081 and 202110559097 to HZ); and Flagship Specialty Construction Project General Surgery (Funding no. 711003).

## Acknowledgments

We acknowledge the members of H. Zhang’s laboratory for their technical help and discussion.

## Conflict of interest

The authors declare that the research was conducted in the absence of any commercial or financial relationships that could be construed as a potential conflict of interest.

## Publisher’s note

All claims expressed in this article are solely those of the authors and do not necessarily represent those of their affiliated organizations, or those of the publisher, the editors and the reviewers. Any product that may be evaluated in this article, or claim that may be made by its manufacturer, is not guaranteed or endorsed by the publisher.
