# Supplementary material for: PTPRO-related CD8+ T-cell signatures predict prognosis and immunotherapy response in patients with breast cancer
Source: Front Immunol. 2022 Aug 8;13:947841. doi: 10.3389/fimmu.2022.947841 (PMC9393709; doi:10.3389/fimmu.2022.947841)
Supplement: Supplementary file 1 [file DataSheet_1.docx]

# Supplementary Information

**Supplementary Figures and Figure legends**

**
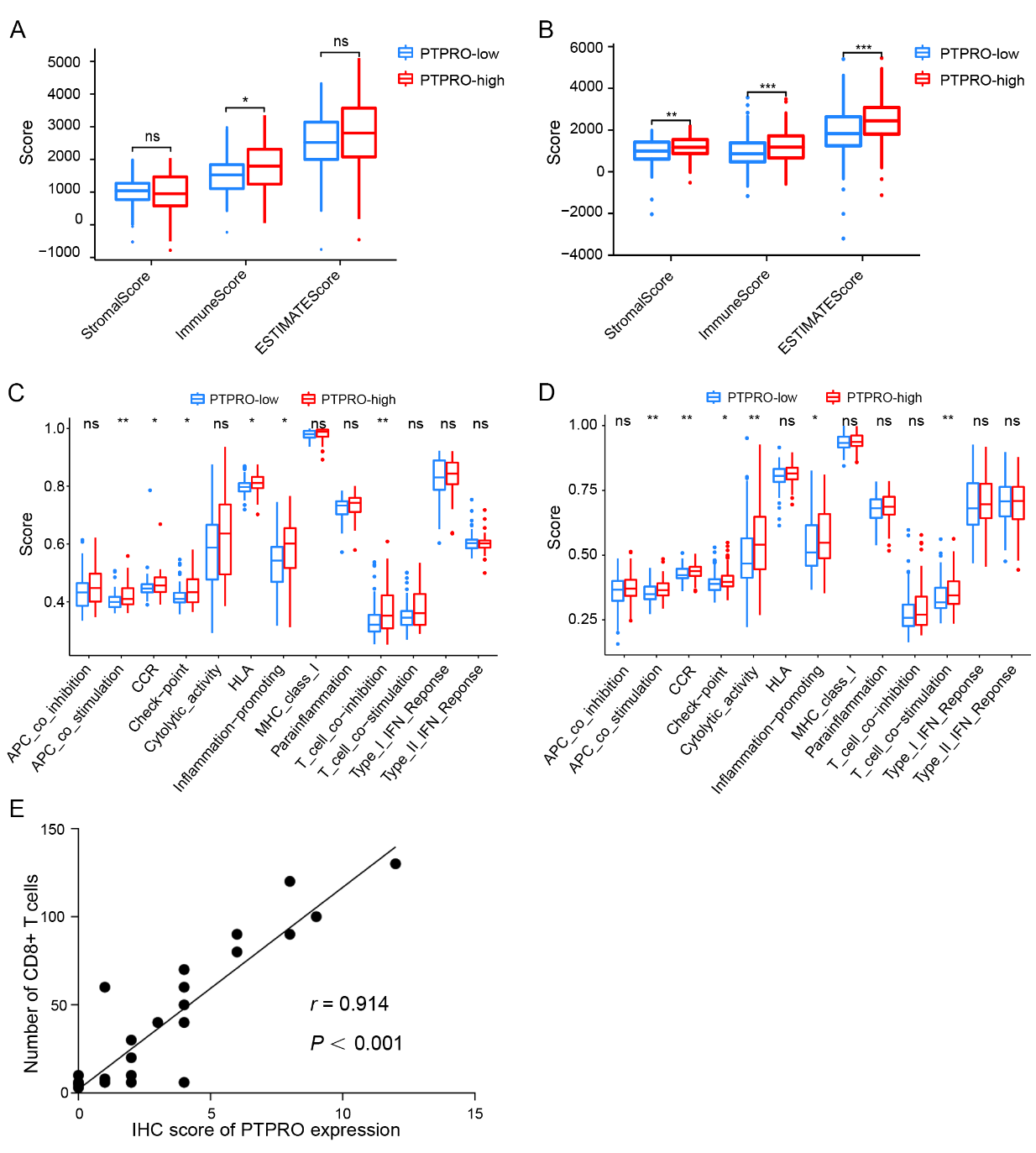
**

**Figure S1**. **Function of PTPRO in breast cancer tumor microenvironment**. Distribution of stromal score, immune score, and estimate score in PTPRO-high and PTPRO-low groups using the ESTIMATE algorithm in GSE65194 (**A**) and GSE3494 (**B**). Comparison of the ssGSEA scores about immune-relatived functions between PTPRO-high group and PTPRO-low group in GSE65194 (**C**) and GSE3494 (**D**) cohorts. (**E**) Pearson’s correlation analysis between the number of CD8^+^ T and IHC score of PTPRO expression in 30 human breast cancer specimens.

**
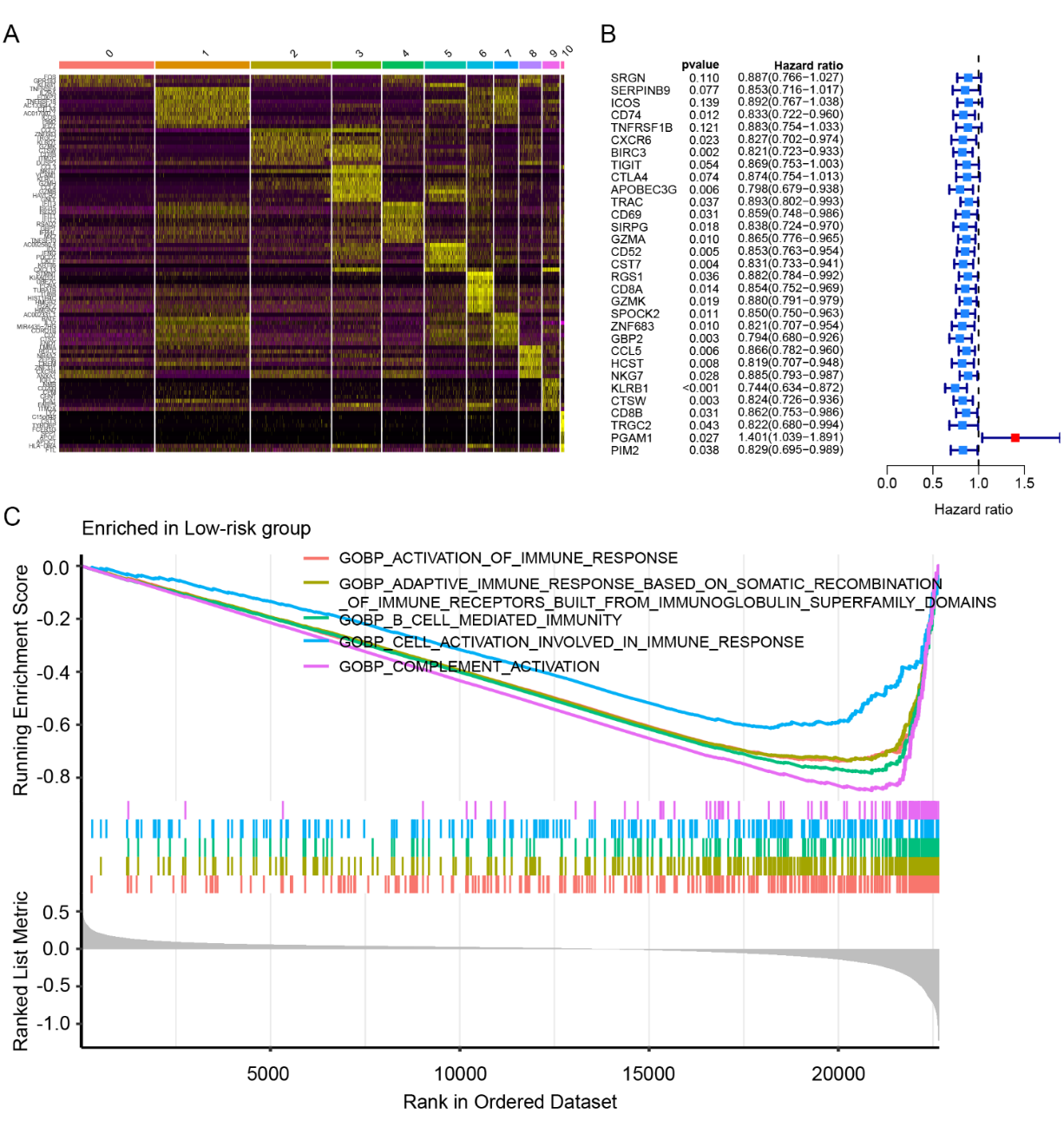
**

**Figure S2| Construction of PTS and GSEA analysis in the training set.** (**A**) Identification marker genes of different cell types. (**B**) Candidate genes of prognostic signature were determined by univariate analysis in the training set. (**C**) GSEA plot depicting immune-related gene sets identified enriched in patients with low risk.


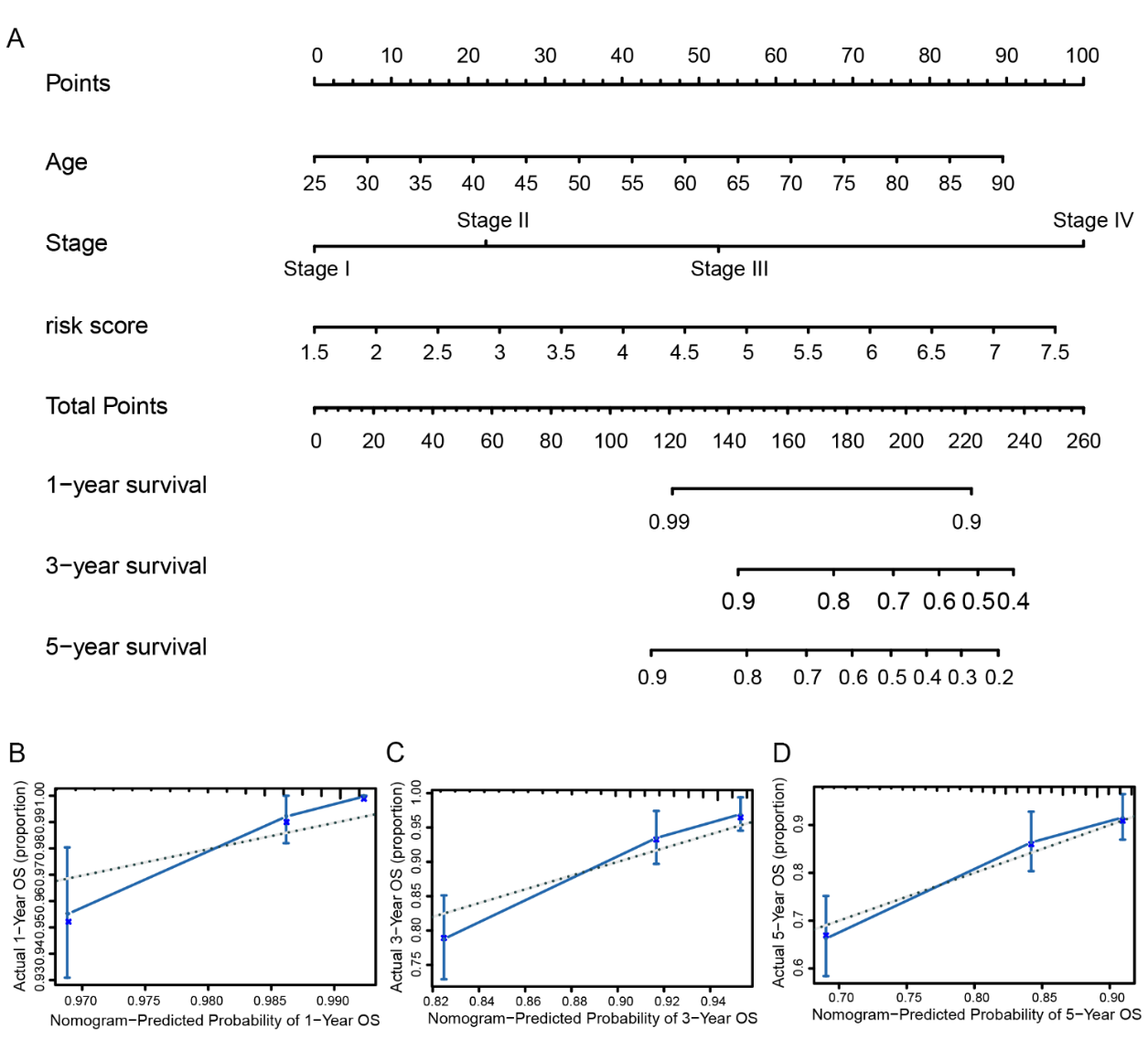


**Figure S3| Nomogram to predict prognostic probabilities in the TCGA training dataset.** (**A**) Nomogram for predicting 1-, 3-, and 5-year OS of breast cancer patients. (**B–D**) Calibration curves of 1-, 3-, and 5-year OS of breast cancer patients.


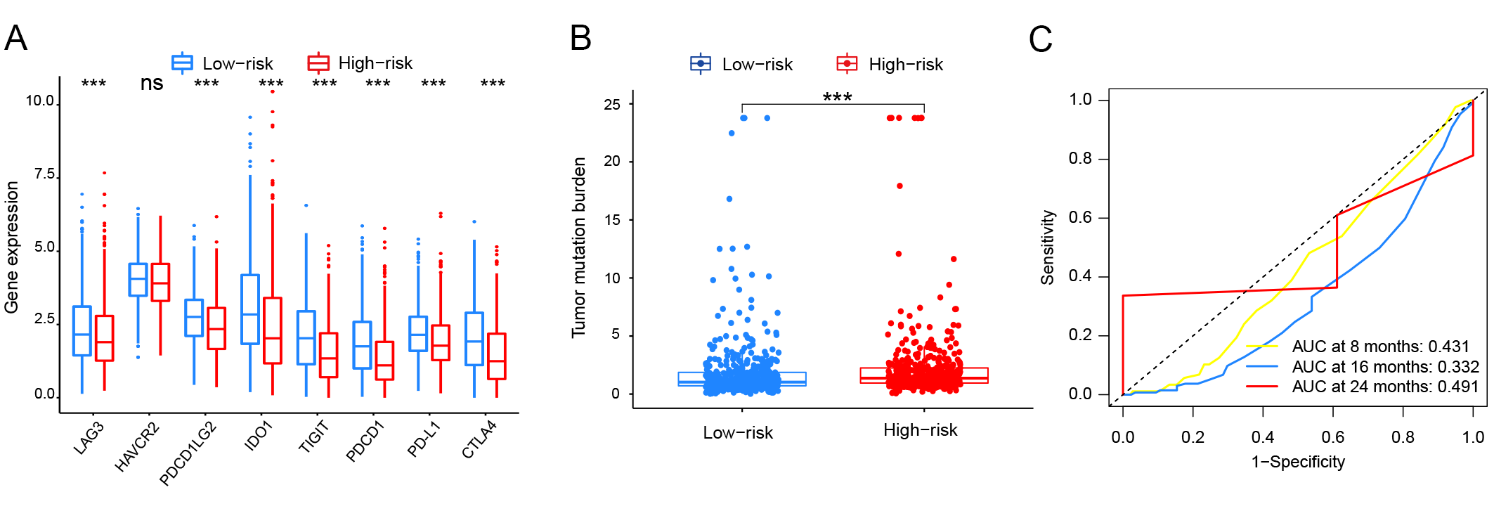


**Figure S4|** TMB was not an efficiency tool for immunotherapy response prediction**.** (**A-B**) Boxplot depicted the distribution of checkpoint-related genes and TMB in different PTS subgroups. (**C**) Receiver-operating characteristic (ROC) curves evaluating the predictive value of the TMB in the IMvigor210 cohort.
